# Genome-Wide Identification and Expression Profiling of the *Di19* Gene Family in Sweet Potato and Its Two Diploid Relatives

**DOI:** 10.3390/genes17060712

**Published:** 2026-06-21

**Authors:** Zitong Yang, Jiaquan Pan, Sitong Liu, Tao Yu

**Affiliations:** Crop Research Institute, Liaoning Academy of Agricultural Sciences, Shenyang 110161, China; yangzitong9870@163.com (Z.Y.); pjqamy1001@163.com (J.P.); 15909824109@163.com (S.L.)

**Keywords:** sweet potato, *Di19*, tissue expression, hormone treatment, biotic and abiotic stresses

## Abstract

**Background:** Di19 (drought-induced 19)proteins belong to the C2H2-type zinc-finger family and play a crucial role in regulating plant growth, developmental processes, hormone signal transduction, and abiotic stress adaptation. However, research on the *Di19* gene family in sweet potato and its diploid relatives remains relatively limited. **Methods**: At the whole-genome level, members of the *Di19* gene family in sweet potato (*Ipomoea batatas*, 2*n* = 6*x* = 90) and its two diploid relatives, *Ipomoea trifida* (2*n* = 2*x* = 30) and *Ipomoea triloba* (2*n* = 2*x* = 30) were systematically identified, and multi-dimensional bioinformatics analyses were carried out. **Results**: Seven *Di19* genes were identified per species, with the family’s overall evolutionary characteristics conserved. Some IbDi19s showed species-specific structural variations, mainly manifested as an increase in the number of exons, loss or substitution of conserved motifs. The expression patterns of *Di19s* of two diploid relatives are highly conserved. *IbDi19s* are mainly expressed in leaves and roots. Most members respond significantly to JA treatment, but hardly respond to IAA. The expression of *IbDi19-1* was significantly up-regulated by 336-fold and 68-fold under GA3 and cold treatments, respectively. Based on bioinformatics and expression data, a hypothesis was proposed that *IbDi19-1* may be involved in the regulation of low-temperature response and gibberellin signaling pathways. **Conclusions**: This study provides candidate genes and a theoretical basis for evolutionary analysis, stress-resistant molecular breeding of the *Di19* gene family in sweet potato and its two diploid relatives.

## 1. Introduction

Sweet potato (*I. batatas* (L.) Lam.) is a hexaploid tuberous-rooted crop belonging to the genus *Ipomoea* in the Convolvulaceae family. It serves multiple purposes, functioning as food, feed, industrial raw material, and having health-care benefits [1]. It holds a significant position in global food security, nutrition improvement, and sustainable agriculture [2]. Sweet potato is widely cultivated in tropical and subtropical regions [3]. Its tuberous roots are rich in carbohydrates, protein, vitamins, minerals, and antioxidant components, and thus it is regarded as a superfood [4,5,6]. Sweet potato is characterized by high and stable yields. Naturally, it has multiple excellent stress-resistant properties, such as tolerance to poor soil, salinity, drought, heavy-metal enrichment, and pesticide residues, highlighting its significant value in breeding [7]. Systematic identification of key functional genes related to stress tolerance can provide a core genetic resource foundation for sweet potato genetic improvement, including transgenic germplasm creation and marker-assisted breeding. With the rapid development of sequencing technology, the complete genomes of sweet potato and its diploid relatives have been successfully assembled, which provides an accurate genomics reference for deciphering the genetic basis of key agronomic traits, mapping candidate genes, and elucidating molecular regulatory pathways [8,9].

Throughout the entire growth cycle of plants, they are confronted with a multitude of extreme and recurrent internal and external survival stresses. For instance, cold, drought, salinity–alkalinity, high temperature, waterlogging, pest infestations, viral infections, chemical residues, oxidative stress, etc., constantly pose threats to the normal growth and development of plants, restrict agricultural production, and in severe cases, may even cause plant death [10]. To adapt to diverse environmental stresses, plants modulate functional gene transcription via complex signaling cascades, triggering a set of physiological and biochemical responses that assemble a coordinated signal transduction network for mitigating stress-triggered plant injury [11]. Among these, transcriptional regulation plays a dominant role in the process of plant stress responses [11].

Zinc-finger proteins are one of the major transcription factor families in eukaryotes. The classical zinc-finger domain enables it to interact with or bind to DNA, RNA, or other proteins, participating in plant growth and development as well as the regulation of responses to abiotic stresses such as salinity, drought, flooding, low temperature, and oxidative stress [12,13]. The *Di19* (drought-induced protein 19) family belongs to a class of atypical Cys2/His2 (C2H2)-type zinc-finger proteins, which contain two C2H2 zinc-finger domains [14]. The *Di19* transcription factor family has typical conserved structural features: its N-terminus contains a highly conserved domain of approximately 50 amino acids, zf-Di19, which is composed of two tandem C2H2 zinc-finger motifs, zinc-finger 1 and zinc-finger 2, while the C-terminus has a highly conserved domain of about 110 amino acids, Di19_C, and there is usually also a nuclear localization signal NLS between the two domains [15]. The Di19 gene family has been identified in many species. Specifically, 7, 7, 18, 7, 16, 15, 16, 7, 10, 6, and 7 family members have been identified in *Arabidopsis thaliana*, *Oryza sativa*, wheat, maize, cotton, soybean, peanut, barley, moso bamboo, grapevine and poplar, respectively [14,16,17,18,19,20,21,22,23,24,25].

In *A. thaliana*, BAPID interacts with both BRM and Di19 simultaneously under drought stress and binds to the promoter regions of *PR1/2/5* genes, relieving the inhibitory effect of BRM on Di19 [26]. This enhances the nucleosome accessibility of PR gene loci by Di19, and Di19 promotes the expression of PR genes, positively regulating the plant’s drought response [26]. Moreover, this expression-enhancing effect can be strengthened by CPK11, which interacts with Di19 in the cell nucleus [27]. AtDi19-3 interacts with IAA14, accelerating the degradation rate of the IAA14 protein, which positively activates the auxin signaling pathway, effectively promoting the normal initiation and development of lateral roots in *A. thaliana* [28]. Additionally, AtDi19-3 can also mediate the auxin-ethylene cross-regulation, jointly controlling lateral root development [28]. Meanwhile, *AtDi19-3* serves as a negative regulator of genes related to auxin synthesis, preventing excessive accumulation of IAA and maintaining a stable balance of auxin content in plants [28]. *AtDi19-3* and *IAA14* jointly participate in the response to abiotic stresses, and the T-DNA insertion mutant of *AtDi19-3* exhibits higher drought and salt tolerance as well as lower ABA sensitivity compared to the wild-type [28]. Another study indicates that *AtDi19-3* is induced for expression by NACl, mannitol, and ABA, and the T-DNA insertion mutation of *AtDi19-3* leads to an increase in the plant’s tolerance to drought, high salinity stress, and ABA, while over-expression of *AtDi19-3* presents an opposite phenotype [29].

In common beans, RT-qPCR analysis shows that different members of the *PvDi19* gene family are induced for expression to varying degrees under PEG treatment, salt stress, cold stress, and cadmium treatment [30]. In soybean, GmDi19-5 interacts with GmLEA3.1 [31]. Over-expression of the negative regulator *GmDi19-5* enhances the sensitivity of transgenic *A. thaliana* to salinity, drought, oxidative stress, and ABA stress, and it participates in the ABA and SOS signaling pathways by altering the transcription of stress-related genes [31]. GmPUB21 and GmDi19-5 interact to coordinately regulate the drought and salt tolerance of soybean through the ABA-dependent pathway [32]. Over-expression of *GmDi19-5* leads to hypersensitivity to drought and high salinity in transgenic plants, while silencing of *GmDi19-5* enhances drought and salt tolerance [32]. *GmDi19-5* is induced for expression by high temperature and may interact with GmDnaJ to perform related functions [33]. The *GmDi19-15* gene is specifically expressed in roots, and over-expressing *GmDi19-15* in *A. thaliana* reduces its drought tolerance [20]. In the transcriptome analysis of *GmDi19-15* over-expressing transgenic plants and wild-type plants, multiple differentially expressed genes are annotated to the auxin signaling pathway [20]. The promoter of *GmDi19-3* plays a crucial role in the responses of vegetable soybean to salt stress and exogenous ABA and MeJA signals [34].

In wheat, upon over-expressing *TaDi19-7* in *A. thaliana*, the transgenic plants display higher sensitivity to salt and drought stresses than the wild-type plants [17]. Moreover, the over-expression of *TaDi19-7* also facilitates the flowering and branching of the transgenic *A. thaliana* [17]. *TaDi19A* was identified from wheat transcriptomic data under cold stress, and its transcription is strongly activated upon cold exposure [35]. When subjected to cold treatment, *A. thaliana* lines overexpressing *TaDi19A* exhibit longer roots and enhanced cold tolerance relative to wild-type controls [35]. Moreover, this gene may enhance the cold tolerance of transgenic plants by regulating the expression of downstream genes related to cold-stress response [35]. Heterologous over-expression of *TaDi19A* in *A. thaliana* increases the plant’s sensitivity to salinity, ABA, and mannitol at the germination stage [36].

In *O. sativa*, *OsDi19s* are differentially expressed in vegetative tissues and are insensitive to ABA [16]. *OsDi19-3* and *OsDi19-4* are up-regulated after high-salt and drought stresses, and overexpression of *OsDi19-4* may enhance its tolerance to drought stress by strengthening the ROS-scavenging capacity [16]. Downstream of *OsCDPK14*, *OsDi19-4* positively regulates the ABA response by modulating the expression of *O. sativa* ABA-responsive genes *OsASPG1* and *OsNAC18* [37]. In the water-deficiency experiment, transgenic *O. sativa* overexpressing *OsDi19-3* exhibited higher drought tolerance and more significant stomatal closure compared to the wild-type [38].

In maize, the promoter of *ZmDi19-5* can be significantly induced by drought stress, and multiple drought-responsive genes show strong binding activity to the core region of its promoter [18]. Over-expression of *ZmDi19-5* can enhance the drought tolerance of maize and delay the flowering time, while its interaction with ZmFKF1b weakens the expression of downstream related target genes [39]. Overexpressing *ZmDi19-1* in *A. thaliana* enhances the salt tolerance of the transgenic *A. thaliana* plants [40]. *ZmDi19-7* regulates the height of maize plants and the size of their organs by modulating the cell size in maize [41].

In cotton, *GhDi19-1* and *GhDi19-2* are likely downstream targets of CDPK kinase in the ABA signaling pathway, and they regulate their activities through the phosphorylation of serine residues in response to high-salinity stress and ABA signals [42]. The promoters of *GhDi19-1* and *GhDi19-2* are induced by NaCl and mannitol, and the transgenic *A. thaliana* seedlings overexpressing *GhDi19-1* and *GhDi19-2* exhibit hypersensitivity to high salinity and ABA [43]. *GhDi19-3* and *GhDi19-4* may negatively regulate salt stress through the calcium signaling and ABA signaling pathways [19].

In poplar, overexpressing *PtDi19-2* and *PtDi19-7* in *A. thaliana* can enhance the ABA sensitivity and drought tolerance of the transgenic *A. thaliana*; they may play a role in improving the drought tolerance of transgenic plants through the ABA-dependent signaling pathway [25]. In cassava, *MeDi19-1* exhibits tissue-specific expression, mainly exerting regulatory functions in leaves, leaf mid-veins, and storage roots [44]. In peanut, overexpressing *AhDi19-3B* in *A. thaliana* can enhance its drought tolerance by improving water retention and reducing oxidative damage [21].

The functions of *Di19* genes in model plants and major crops such as *A. thaliana*, soybean, *O. sativa*, wheat, maize, and cotton have been reviewed previously. Numerous studies have confirmed that *Di19* is a highly conserved stress-tolerant transcription factor, playing an important regulatory role in responses to drought, low temperature, and salt stress, as well as in growth and development. It possesses high application value in gene transformation and molecular breeding. Currently, there are no reports at home and abroad on the systematic identification of the *Di19* gene family and research on gene functions in sweet potato and its related species. Considering the special polyploid evolution of sweet potato, the specialized properties of its tuberous root development, and the practical need for stress-resistance improvement in the industry, there is an urgent need to conduct a systematic exploration and analysis of the *Di19* gene family in the genus *Ipomoea*. This will help improve the stress-resistance regulation theory of tuberous root crops and, simultaneously, reserve key gene resources for the stress-resistance genetic improvement of sweet potato.

In summary, this study will, for the first time, systematically identify members of the *Di19* gene family in sweet potato and its two diploid relatives. A comprehensive analysis will be carried out on their chromosomal localization, protein physicochemical characteristics, phylogeny, gene structure, promoter cis-elements, protein–protein interaction networks, and intra- and inter-species collinearity. Additionally, expression pattern analysis will be performed on tissues, exogenous hormones, biotic and abiotic stress treatments.

## 2. Materials and Methods

### 2.1. Identification of Di19 Gene Family Members in Sweet Potato and Its Two Diploid Relatives

To ensure the accuracy of the results, this study employed two methods to identify members of the *Di19* gene family in sweet potato. The complete identification process and screening criteria are detailed as follows.

(1) Genome Data Download

Download the genome sequence file of the pasi3 version of sweet potato (Ipomoea_batatas_pasi3.fa), the entire protein sequence file (Ipomoea_batatas_pasi3.pep.fa), and the gene structure annotation file (Ipomoea_batatas_pasi3.gff3) from the Ipomoea Genome Hub (https://sweetpotao.com, accessed on 5 December 2025).

(2) Acquisition of Query Sequences for Homology Search

Collect the gene IDs of the *Di19* family (AtDi19s, OsDi19s) from published literature in model plants *A. thaliana* and *O. sativa* [14,16]. Download the corresponding complete protein sequences (Arabidopsis_thaliana.TAIR10.pep.all.fa; osa1_r7.all_models.pep.fa) from the TAIR10 database and the Rice Genome Annotation Project database, respectively. Extract the above-mentioned reference protein sequences to serve as the homology search set.

(3) Screening via BLASTP Homology Search

Using the Di19 protein sequences of *A. thaliana* and *O. sativa* as queries, BLASTP alignment was performed against all the protein sequences of sweet potato via TBtools (v2.305) [45]. The screening thresholds were set as follows: E-value ≤ 1 × 10^−5^, sequence identity ≥ 30%, and Subject coverage ≥ 50%. Consequently, a preliminary set of homologous candidate proteins was obtained.

(4) Screening by HMMER Domain Search

Download the HMM model files corresponding to the characteristic domains of the Di19 family, zf-Di19 (PF05605) and Di19_C (PF14571), from the Pfam database (http://pfam.xfam.org/, accessed on 5 December 2025). Use the HMMER within TBtools (v2.305) to batch-search the sweet potato protein sequences [45]. The screening criterion is an E-value ≤ 1 × 10^−5^, and only candidate proteins with domain-matching signals are retained.

(5) Filtration of Redundant Sequences and Alternative Splicing Isoforms

Merge the candidate protein lists obtained from the above two methods. Locate the gene locus corresponding to each protein according to the sweet potato annotation file. When multiple alternative splicing transcripts of the same gene encode multiple protein isoforms, retain only the protein sequence of the main transcript with the longest coding sequence. For multiple entries of the same gene repeatedly hit by the two methods, merge and remove redundancy, keeping only one representative protein sequence for each gene.

(6) Domain Verification and Final Member Determination

Submit the redundancy-removed candidate proteins in batches to the CD-search online tool (https://www.ncbi.nlm.nih.gov/Structure/wrpsb-out/wrpsb.cgi, accessed on 10 December 2025) for conservative domain verification. A protein must contain both the zf-Di19 and Di19_C conserved domains intact. If either domain is absent or the domain-alignment coverage is less than 70%, the protein is considered to have a defective domain and is directly eliminated.

After a step-by-step screening and verification process, the members of the *Di19* gene family in sweet potato were finally determined.

The identification of *Di19* gene family members in the two diploid relatives follows an analysis procedure completely identical to that of sweet potato. Full-set omics data of *I. triloba* and *I. trifida* were obtained from the Sweetpotato Genomics Resource (https://sweetpotato.uga.edu/gt4sp_download.shtml, accessed on 10 December 2025). The data included genome sequence files (*Ipomoea trifida* (NSP306) Hard Masked Genome Assembly (v3).fa; *Ipomoea triloba* (NSP323) Hard Masked Genome Assembly (v3)).fa, entire protein sequence files (NSP323_*triloba*_v3.hc.gene_models.pep.fa; NSP306_*trifida*_v3.hc.gene_models.pep.fa), and gene structure annotation files (NSP323_*triloba*_v3.hc.gene_models.gff3; NSP306_*trifida*_v3.hc.gene_models.gff3).

### 2.2. Analysis of Protein Physicochemical Properties

The genomic length, CDS length, and number of amino acids of each IbDi19 were summarized through genomic files. The molecular weight, theoretical pI, instability index, and hydrophilicity of IbDi19 proteins were calculated on ExPASy (https://www.expasy.org/, accessed on 28 February 2026). The subcellular localization of IbDi19s proteins was predicted on Plant-mPLoc (http://www.csbio.sjtu.edu.cn/bioinf/plant-multi/, accessed on 28 February 2026).

### 2.3. Chromosome Localization

The chromosomal location information of *IbDi19s* was retrieved from the sweet potato genome annotation file (Ipomoea_batatas_pasi3.gff3). Visualize the chromosomal localization of the genes using the software TBtools (v2.305) [45].

### 2.4. Phylogenetic Analysis

First, the protein sequences of *A. thaliana*, *O. sativa*, sweet potato, and its two relatives were aligned using MAFFT version 7 (https://mafft.cbrc.jp/alignment/server/, accessed on 8 March 2026). The maximum-likelihood (ML) phylogenetic tree was constructed using PhyML 3.0 (http://www.atgc-montpellier.fr/phyml/, accessed on 13 June 2026). The LG amino acid substitution model was selected. The evolutionary rate heterogeneity among sites was corrected using a Gamma distribution with four rate categories, and the proportion of invariable sites was estimated automatically by the software. The initial phylogenetic tree was generated using the BioNJ algorithm. Subsequently, both the tree topology and branch lengths were fully optimized. A standard bootstrap test with 1000 replicates was set up to assess the confidence of each branch. A bootstrap support value of ≥700 was used as the threshold for determining the reliability of the branch topology. The phylogenetic tree of IbDi19s, ItbDi19s and ItfDi19s was constructed in the same way. Finally, the phylogenetic tree was beautified on iTOL (https://itol.embl.de/, accessed on 13 June 2026).

### 2.5. Gene Structure Analysis

Obtain the domain information of IbDi19s, ItbDi19s and ItfDi19s on NCBI-CDD (https://www.ncbi.nlm.nih.gov/Structure/cdd/wrpsb.cgi, accessed on 8 March 2026). Conserved Motif prediction analysis was carried out using the MEME tool in TBtools (v2.305). The maximum number of Motifs to be retrieved was set to 8, and the remaining parameters were kept as the default. Combine the phylogenetic tree file and the genomic file, and use TBtools (v2.305) to visualize and analyze the results [45].

### 2.6. Promoter Cis-Acting Element Analysis

Extract approximately 2000 bp of the promoter sequence upstream of the *IbDi19s* genes using TBtools (v2.305) [45]. The cis-acting elements within the sequences were predicted using PlantCARE (https://bioinformatics.psb.ugent.be/webtools/plantcare/html/, accessed on 11 May 2026), and the original copy numbers of each cis-element in every promoter were counted. The original copy numbers of the elements were log2-transformed and then Min–Max normalized to the range of 1.00–3.50. A heatmap was plotted based on these normalized values.

### 2.7. Protein–Protein Interaction Analysis

Based on the Di19 homologous proteins in *A. thaliana*, the protein–protein interaction network of Di19 in sweet potato was predicted using String (https://cn.string-db.org/, accessed on 11 May 2026). The confidence level was set to 0.700. Network visualization was accomplished using Cytoscape software (v3.10.3), with isolated nodes removed during the visualization process. This interaction relationship is merely a predictive result deduced from homologous sequences and has not been verified by in vivo or in vitro experiments.

### 2.8. Collinearity Analysis

For intragenomic collinearity analysis, the Multiple Collinearity Scan toolkit (MCScanX) in TBtools (v2.305) was used to perform collinearity analysis of the genomes of sweet potato and its two diploid relatives against themselves (E-value ≤ 1 × 10^−3^), and then a circos plot was drawn for each species [45]. For interspecies collinearity analysis, the Multiple Collinearity Scan toolkit (MCScanX) was also utilized. Collinearity analyses between *I. trifida* and *I. batatas*, as well as between *I. batatas* and *I. triloba*, were carried out separately. Subsequently, the collinearity information of the three species was merged and visualized. The CDS sequences and protein sequences corresponding to the collinear gene pairs obtained from intra-and inter-species collinearity analyses were extracted. Selection pressure evolutionary analysis was carried out to calculate the Ka/Ks values by using TBtools (v2.305) software [45].

### 2.9. Expression Analysis

#### 2.9.1. Quantitative Expression Analysis of Sweet Potato via RT-qPCR

(1) Experimental Materials and Sampling Design

Samples were taken from the roots, stems, and leaves of test-tube seedlings of Liaoshu 40 that had grown for 30 days. After transplanting the test-tube seedlings of Liaoshu 40 into nutrient soil and culturing for 4 days, four treatments were set up: cold treatment at 4 °C, 100 μM GA3, 100 μM IAA, and 100 μM JA. Samples were taken at 0 h, 1 h, 3 h, 6 h, 12 h, and 24 h, respectively. Each sampling group was set up with three independent biological replicates. For the cDNA sample of each biological replicate, three technical replicates of qPCR were performed.

(2) RT-qPCR Reaction System and Primers

The primer sequences for RT-qPCR analysis are listed in Table 1.

(3) Calculation Method of Relative Expression Level

*IbActin* was selected as the reference gene, and the relative expression levels of the target genes were calculated using the classic 2^−ΔΔCt^ method. The data of individual samples were calibrated using the reference gene. With the expression level of the control group set as 1, inter-group normalization was performed. All treatment and tissue samples were converted into expression multiples relative to the control group.

(4) Statistical Analysis and Graphing

Both statistical analysis and graphing were accomplished using GraphPad Prism (v10.1.2) software. For comparisons among multiple groups, One-way ANOVA and Tukey’s multiple-comparison test were employed, with a significance threshold of *p* < 0.05. In the bar chart, different lowercase letters denote statistically significant differences between groups.

#### 2.9.2. RNA-Seq Expression Analysis of Diploid Relatives

In this study, the expression levels of *ItbDi19s* and *ItfDi19s* were investigated in different tissues, under different hormone treatments, treatments with Beta-Aminobutyric Acid and benzothiadiazole S-methylester, as well as treatments with mannitol and NaCl. The treatment concentrations of ABA, BAP, GA_3_, and IAA were 50 μM, 10 μM, 50 μM and 10 μM, respectively. For the low-temperature stress, the control group was cultured at a daytime temperature of 28 °C and a nighttime temperature of 22 °C. The treatment group was exposed to a daytime temperature of 10 °C and a nighttime temperature of 4 °C. Regarding the high-temperature stress, the control group was cultivated under the condition of 28 °C during the day and 22 °C at night. The treatment group was subjected to a constant high-temperature treatment of 35 °C day and night. The gene expression levels were represented by Fragments Per Kilobase of exon model per Million mapped reads (FPKM). The FPKM values were transformed using log_2_(FPKM), and a heatmap was drawn with the TBtools (v2.305) software.

The RNA-seq expression data of the two diploid relatives were both sourced from the public data platform of the GT4SP (Genomic Tools for Sweetpotato Improvement) project, specifically the Sweetpotato Genomics Resource (https://sweetpotato.uga.edu/gt4sp_download.shtml, accessed on 7 May 2026). The expression matrix files used were: I_*triloba*_NSP323_FPKM_expression_matrix_v3_anno.xlsx; I_*triloba*_NSP323_stress_FPKM_expression_matrix_v3_anno.xlsx; I_*trifida*_NSP306_FPKM_expression_matrix_v3_anno.xlsx; I_*trifida*_NSP306_stress_FPKM_expression_matrix_v3_anno.xlsx.

The expression matrix is generated from the original RNA seq sequencing reads in NCBI BioProject corresponding to *I. trifida* and *I. triloba*, using both double-ended and single-stranded library modes. Tophat (v2.1.0) was used to align them to the respective v3 version reference genome assembly sequences, and then the FPKM expression levels were calculated using Cufflinks (v2.2.1) to generate the dataset for the v3 high-confidence gene model. The public identifiers of their upstream raw data are: *I. triloba*: PRJNA428241, SRP162110; *I. trifida*: PRJNA428214, SRP162021. Meanwhile, the complete data of the GT4SP project have been archived in the Dryad database [46]. The relevant data can be publicly accessed through the above-mentioned identifiers and the official website of the project.

## 3. Results

### 3.1. Identification and Characteristic Analysis of Di19 Gene Family Members in Sweet Potato and Its Two Diploid Relatives

**Figure 1:**
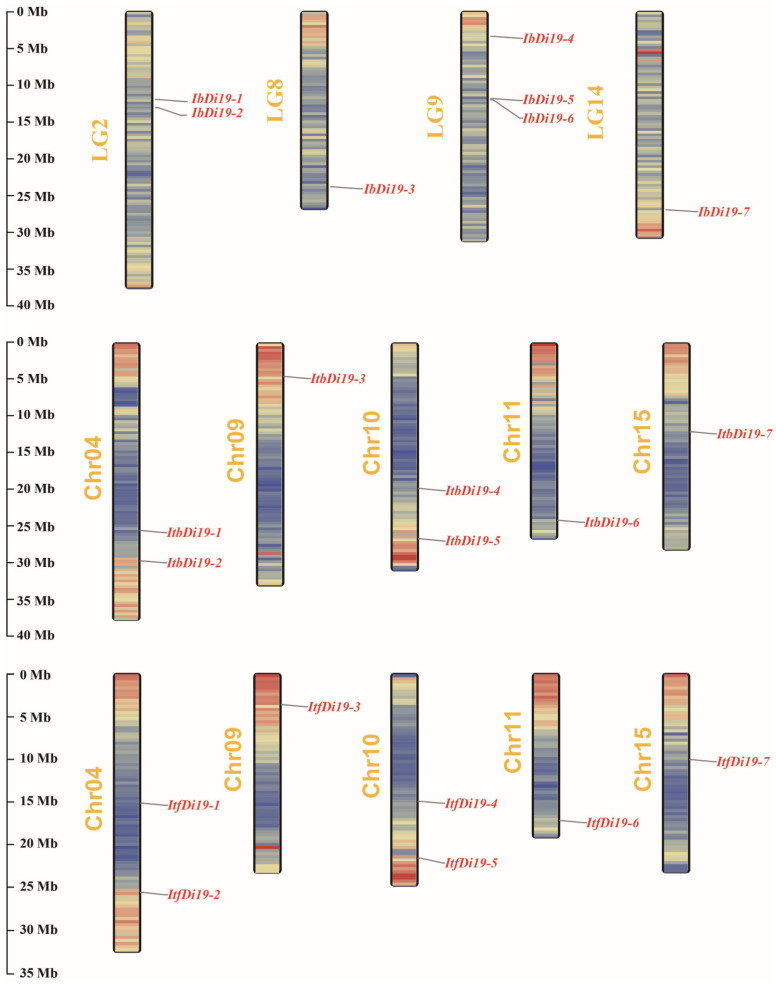
Localization of *Di19s* on the chromosomes of sweet potato and its two diploid relatives. The chromosomal color gradient from blue to red represents ascending gene density.

In this study, methods such as Blastp, hmmersearch, and CD-search were comprehensively employed, and seven members were identified in sweet potato and its two diploid relatives (Figure 1). They were named *IbDi19-1* to *IbDi19-7*, *ItbDi19-1* to *ItbDi19-7*, and *ItfDi19-1* to *ItfDi19-7* in sequence according to their positions on the chromosomes. The chromosomal localization numbers and physical segment distributions of *Di19s* in the two diploid relatives are highly conserved. In contrast, the chromosomal distribution pattern of *IbDi19s* shows significant divergence. In sweet potato, *IbDi19s* are distributed on 4 chromosomes. Two are located on LG2, one on LG8, three on LG9, and one on LG14. *Di19s* in both diploid relatives are distributed across five chromosomes. Two are on Chr04, one on Chr09, two on Chr10, one on Chr11, and one on Chr15.

The sequence characteristics and the physicochemical properties of IbDi19s were analyzed (Table 2). The genomic length of IbDi19s ranged from 2425 bp (IbDi19-7) to 4243 bp (IbDi19-5), and the CDS length ranged from 507 bp (IbDi19-2) to 975 bp (IbDi19-3). The amino acid length of IbDi19s was from 168 aa (IbDi19-2) to 324 aa (IbDi19-3), and their molecular weight ranged from 19.06 kDa (IbDi19-2) to 36.06 kDa (IbDi19-3). The isoelectric point of IbDi19s ranged from 4.45 (IbDi19-2) to 6.53 (IbDi19-3), all of which were less than 7, indicating that they were acidic proteins. The instability index of all IbDi19s proteins was greater than 40, suggesting that these proteins were unstable in vitro. The GRAVY scores of all IbDi19s were less than 0, indicating that they were all hydrophilic proteins. Among them, IbDi19-2 had the strongest hydrophilicity, and IbDi19-7 had the weakest hydrophilicity. The results of subcellular localization prediction showed that IbDi19s were all distributed in the nucleus.

### 3.2. Phylogenetic Analysis of Di19 Proteins

**Figure 2:**
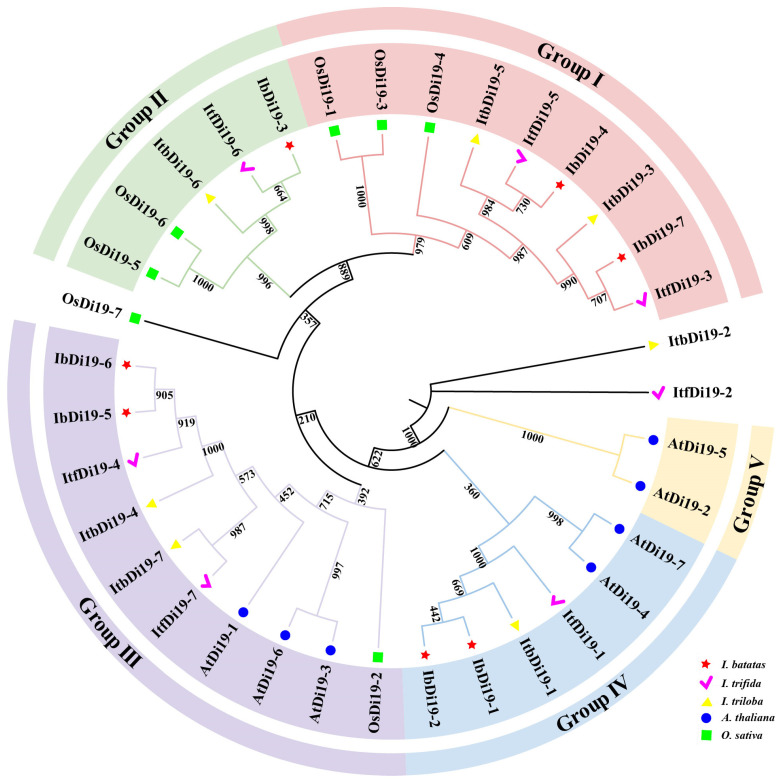
Phylogenetic tree of Di19 proteins. The pink band represents Group I, the green band represents Group II, the purple band represents Group III, the blue band represents Group IV, and the yellow band represents Group V. Different shapes represent different species. The blue circle represents *A. thaliana*, the green square represents *O. sativa*, the red five-pointed star represents *I. batatas*, the yellow triangle represents *I. triloba*, and the purple check mark represents *I. trifida*. The numbers on the branches of the phylogenetic tree are bootstrap values.

A phylogenetic tree of 35 Di19 proteins from five species, namely *A*. *thaliana*, *O*. *sativa*, *I*. *batatas*, *I*. *triloba*, and *I*. *trifida*, was constructed based on the maximum-likelihood method (Figure 2). The tree was divided into five major evolutionary branches (Group I~Group V), containing 9, 5, 10, 6, and 2 members, respectively. OsDi19-7, ItbDi19-2, and ItfDi19-2 were not classified into the above-mentioned five branches. In terms of species composition, Group V was a branch specific to *A*. *thaliana*, containing only two members, AtDi19-2 and AtDi19-5. The remaining four branches (Group I~Group IV) all showed cross-species distribution characteristics, but did not cover all five studied species.

The Di19 protein sequences of sweet potato and its two diploid relatives are highly conserved, showing distinct clustering characteristics in each evolutionary branch. In Group I, IbDi19-4, ItfDi19-5, and ItbDi19-5 cluster closely together. In Group III, IbDi19-5, IbDi19-6, ItfDi19-4, and ItbDi19-4 are tightly clustered. Some AtDi19s are located outside the clustering lineages of sweet potato and its two diploid relatives. For instance, in Group III, AtDi19-1, AtDi19-3, and AtDi19-6, and in Group IV, AtDi19-4 and AtDi19-7. Most OsDi19s either cluster outside the dicot species or form a monocot-specific sub-cluster alone. For example, in Group I, OsDi19-1, OsDi19-3, and OsDi19-4. In Group II, OsDi19-5 and OsDi19-6. In Group III, OsDi19-2. Bootstrap support values for most nodes of closely related sequences are greater than 700. Only some nodes of distantly related branches have relatively low support.

### 3.3. Gene Structure Analysis of Di19s in Sweet Potato and Its Two Diploid Relatives

**Figure 3:**
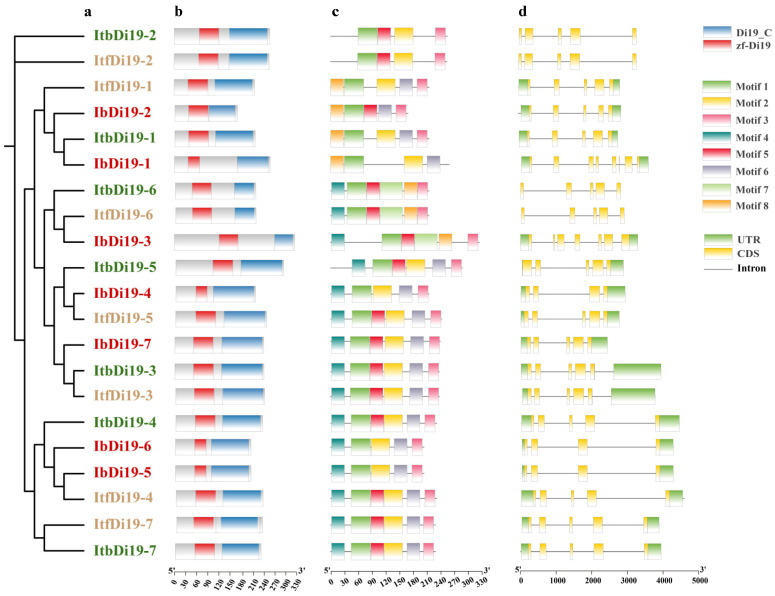
Conserved domains and intron-exon structures of sweet potato and its two diploid relatives. (**a**) Phylogenetic tree of Di19s proteins. (**b**) Analysis of Di19s protein domains. (**c**) Motif analysis of Di19s proteins. (**d**) Analysis of *Di19s* intron-exon structure. Red word font represents IbDi19s, green word font represents ItbDi19s, and brown word font represents ItfDi19s in (**a**). The red block represents the zf-Di19 domain, and the blue block represents the Di19_C domain in (**b**). The green and yellow blocks represent the untranslated regions and exons, respectively, and the gray lines represent introns in (**d**).

The phylogenetic tree of sweet potato and its two diploid relatives reveals that the Di19 protein sequences of the three species are highly conserved. Different gene members have diverged into multiple well-defined evolutionary branches. For example, IbDi19-7, ItbDi19-3, and ItfDi19-3 cluster closely together. IbDi19-4, ItbDi19-5, and ItfDi19-5 form another clustering branch (Figure 3a).

The analysis of the protein domains of Di19s reveals that all Di19s proteins possess Di19_C and zf-Di19 domains (Figure 3b). The zf-Di19 domain is adjacent to the N-terminus, and the Di19_C domain is near the C-terminus.

In the motif prediction analysis, the number of motifs in Di19 proteins of sweet potato and its two diploid relatives ranges from four to six (Figure 3c). IbDi19-7, ItbDi19-3, and ItfDi19-3 cluster in one branch of the phylogenetic tree, and the motifs of these three proteins are highly conserved in both number and structure. In addition, for some Di19 proteins clustered in the same branch, although they are highly conserved between the two diploid relatives, the number and structure of motifs in sweet potato have undergone specific variations. For example, the motifs are conserved between ItfDi19-1 and ItbDi19-1, whereas within the same clade, motif 3 is absent in IbDi19-1, and motif 2 in IbDi19-2 is replaced by motif 5. IbDi19-4 has one less motif 5 compared to ItbDi19-5 and ItfDi19-5 in the same branch. When compared with ItfDi19-4 and ItbDi19-4, both IbDi19-5 and IbDi19-6 lack motif 5.

Analysis of the intron-exon structure reveals that *IbDi19s* contain four to eight exons, while both *ItbDi19s* and *ItfDi19s* contain only five exons (Figure 3d). Some *IbDi19s* show certain differences in exon–intron structure compared to *ItbDi19s* and *ItfDi19s* in the same branches. For example, both *IbDi19-5* and *IbDi19-6* contain only four exons, while *ItbDi19-4*, *ItbDi19-7*, *ItfDi19-4*, and *ItfDi19-7* all contain five exons. *IbDi19-2*, *ItbDi19-1* and *ItfDi19-1* all contain five exons, while *IbDi19-1* contains eight exons. *IbDi19-3* contains seven exons, while *ItbDi19-6* and *ItfDi19-6* contain only five exons. *IbDi19-4* contains four exons, while *ItbDi19-5* and *ItfDi19-5* contain only five exons.

### 3.4. Analysis of Cis-Acting Elements in the Promoters of IbDi19s

**Figure 4:**
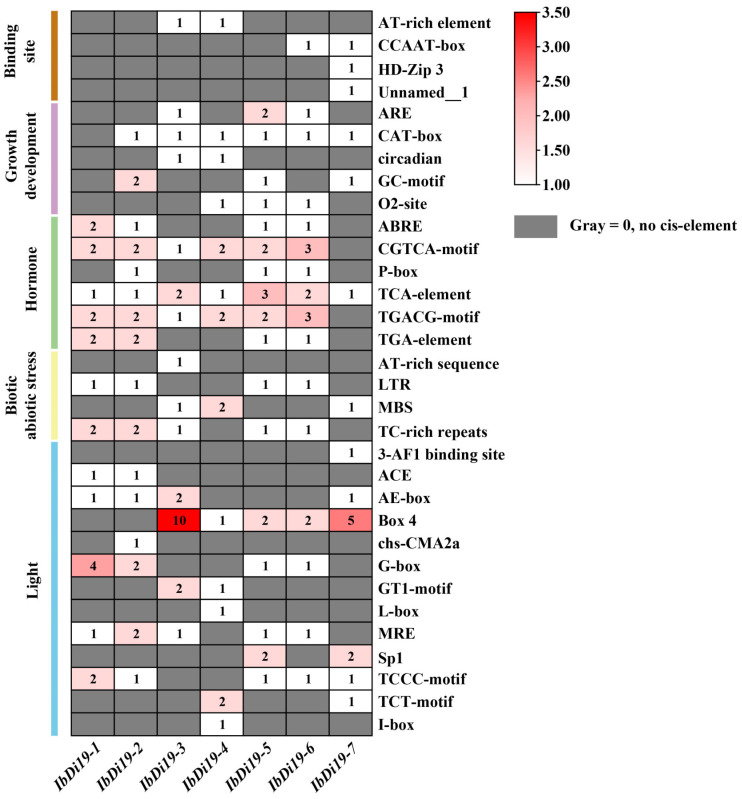
Analysis of cis-acting elements in the promoters of *IbDi19s* in sweet potato. The values within the squares represent the original copy numbers of cis-acting elements. The squares’ colors were assigned according to the log_2_-transformed and normalized values of element counts, with higher values corresponding to darker red. Gray cells represent a value of 0, indicating no corresponding cis-element exists.

We extracted the 2000 bp promoter sequences upstream of *IbDi19s* and conducted an analysis of their cis-acting elements. According to the functional prediction, there are five types of cis-elements within the 2000 bp promoter region upstream of *IbDi19s*, namely binding sites, growth and development elements, hormone-related elements, biotic and abiotic stress-related elements, and light-related elements (Figure 4).

The results showed that all seven *IbDi19s* contain a large number of core promoter elements, common cis-acting elements, and some protein-binding sites. In terms of growth and development, *IbDi19-3*, *-5*, and *-6* contain one, two, and one ARE cis-element(s), respectively, which are essential for anaerobic induction. All except *IbDi19-1* contain one CAT-box cis-element related to meristem expression. *IbDi19-3* and *IbDi19-4* each contain one cis-element involved in circadian rhythm regulation. *IbDi19-2*, *-5*, and *-7* contain two, one, and one enhancer element(s) GC-motif-involved in hypoxia-specific induction, respectively. *IbDi19-4*, *-5*, and *-6* each contain one O2-site cis-element involved in zein metabolism regulation.

In terms of hormones, *IbDi19-1*, *-2*, *-5*, and *-6* contain two, one, one, and one ABRE cis-element(s) involved in abscisic acid response, respectively. All except *IbDi19-7* contain 2~6 cis-elements involved in methyl jasmonate response, such as CGTCA-motif and TGACG-motif. *IbDi19-2*, *-5*, and *-6* each contain one gibberellin-responsive element P-box. All *IbDi19s* contain 1~3 TCA-elements involved in salicylic acid response. *IbDi19-1*, *-2*, *-5*, and *-6* contain two, two, one, and one auxin-responsive element(s), the TGA element, respectively. In terms of biotic and abiotic stress, *IbDi19-3* contains one cis-element AT-rich sequence related to plant disease resistance. *IbDi19-1*, *-2*, *-5* and *-6* each carry one LTR cis-element associated with cold responsiveness. *IbDi19-3*, *-4*, and *-7* contain one, two, and one MYB-binding site(s) involved in drought inducibility, respectively. All except *IbDi19-4* and *IbDi19-7* contain 1~2 TC-rich repeating cis-elements involved in defense and stress responses. Each *IbDi19s* contains a variety of light-responsive elements, such as Box 4, G-box, TCCC-motif, etc.

### 3.5. Analysis of Protein–Protein Interaction Network of IbDi19s

**Figure 5:**
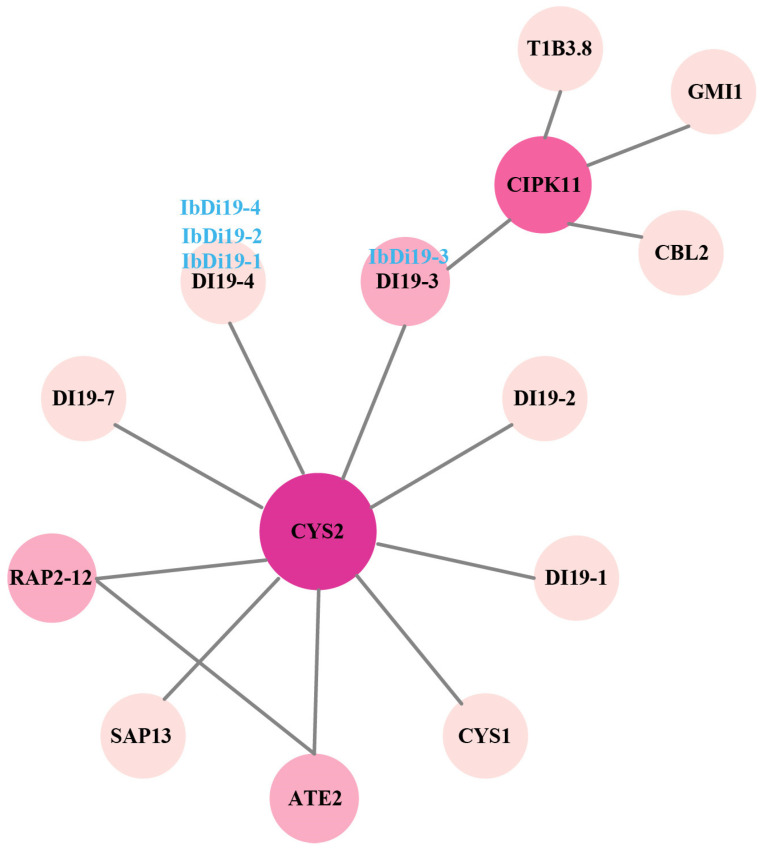
Analysis of the protein–protein interaction network of IbDi19s. The color gradient (from light to dark) and circle size (from small to large) of nodes both correspond to node degree. Darker and larger circles indicate higher node degree, representing more directly interacting proteins. Labels in blue font denote IbDi19 proteins homologous to Di19 proteins of *A. thaliana*.

To explore the potential regulatory network of IbDi19s, we constructed an interaction network predicted based on the Di19 homologous proteins in *A*. *thaliana* (Figure 5). The results showed that the *A*. *thaliana* proteins DI19-1, DI19-2, DI19-3, DI19-4, and DI19-7 interact with four other proteins, RAP2-12, SAP13, ATE2, and CYS1, and all of them interact with CYS2. CYS1 and CYS2 are members of the cystatin gene family, and SAP13 is a member of the stress-associated protein gene family [47,48,49,50]. These proteins are involved in the response and regulation processes of various abiotic stresses such as low temperature, drought, and high salt [47,48,49,50]. The *A*. *thaliana* proteins DI19-3, T1B3.8, GMI1, and CBL2 may interact with CIPK11. As an important protein kinase in the calcium-regulated pathway, CIPK is also involved in the response processes of various abiotic stresses such as low temperature, drought, and salt stress [51,52,53]. Multiple studies have demonstrated that CIPK11 acts as a partial upstream modulator of the drought response in *A*. *thaliana* via targeting the transcription factor Di19-3 [54]. Based on the homologous mapping files, IbDi19 -1, -2, and -4 were determined to be homologous to DI19-4, and IbDi19-3 was homologous to DI19-3 in *A*. *thaliana*. Given these conservation characteristics, it is speculated that homologous proteins in sweet potato may exhibit similar protein–protein interaction patterns. The interaction relationships in this section are all predicted results inferred from homology. They are only used to propose research hypotheses and do not represent the real protein–protein interactions in sweet potato.

### 3.6. Collinearity Analysis of Di19s in Sweet Potato and Its Two Diploid Relatives

**Figure 6:**
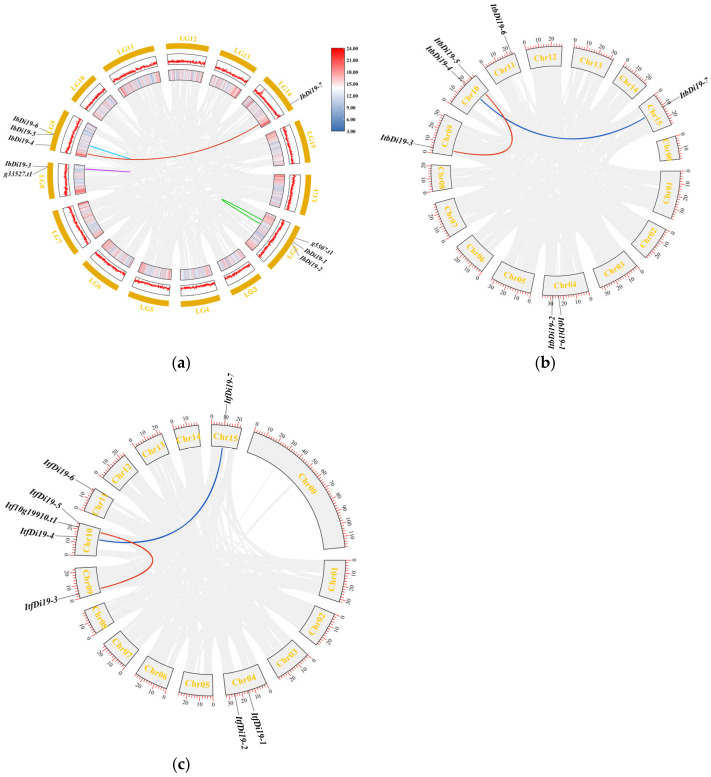
Intraspecific collinearity analysis of sweet potato and its two diploid relatives. (**a**) Intraspecific collinearity analysis of sweet potato. (**b**) Intraspecific collinearity analysis of *I. triloba*. (**c**) Intraspecific collinearity analysis of *I. trifida*. The outermost yellow or gray blocks represent chromosomes, and the scales on the blocks show the length of the chromosomes and the distribution of genes on the chromosomes. Both the red lines in the middle circle and the heatmap in the inner circle in (**a**) represent gene density. The greater the fluctuation of the lines, the higher the gene density. In the heatmap, the color from red to white and then to blue indicates a gradual decrease in gene density. Gray lines show all intra-species collinear genes, and colored lines link *Di19* genes to their collinear genes.

Intraspecific collinearity analysis of *Di19s* genes in sweet potato and its two diploid relatives was carried out. The results showed that *I. batatas*, *I. triloba*, and *I. trifida* contained four, two, and two segmental duplication gene pairs, respectively, with no tandem duplication gene pairs (Table 3 and Figure 6). Among them, *IbDi19-2* and *IbDi19-3* formed segmental duplication gene pairs with *g5507.t1* and *g33527.t1* respectively (Table 3, Figure 6a). These two genes do not belong to the *Di19* gene family, because they both contain only a zf-Di19 domain. Although *itf10g19910.t1* shows collinearity with *ItfDi19-3*, it only harbors a partial Di19_C domain and is not classified as a canonical *ItfDi19* family member.

**Figure 7:**
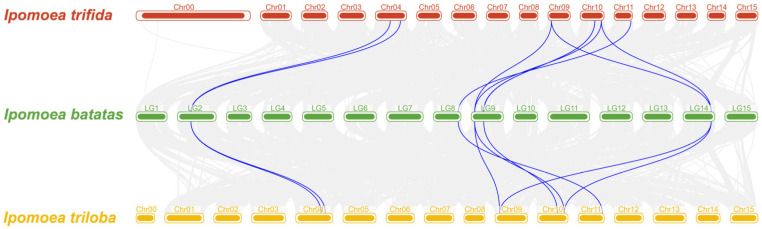
Interspecific collinearity analysis of sweet potato and its two diploid relatives. Blue lines represent collinear links of *Di19* genes between each of the two diploid relatives and sweet potato.

We conducted interspecific collinearity analysis on *A*. *thaliana*, sweet potato, and its two diploid relatives. We found that there were no collinear genes of *Di19s* between *A*. *thaliana* and sweet potato. Therefore, we carried out collinearity gene analysis between the genomes of *I. trifida* and *I. batatas*, and between *I. batatas* and *I. triloba* (Table 4, Figure 7). The results showed that there were eight pairs of gene pairs in each of the two groups of genomes (Table 4 and Figure 7). The collinear genes of *I. batatas* in the two diploid relatives showed a symmetric distribution (Figure 7). *IbDi19-4* and *IbDi19-7* had a collinear relationship with the same gene, *itf10g19910.t1* (Table 4, Figure 7).

Evolutionary selection pressure analyses were performed on the intraspecific and interspecific collinear gene pairs of sweet potato and its two diploid relatives, using the Ka/Ks ratio (Table 5). For some gene pairs, factors such as sequence divergence saturation, absence of substitution sites, and a Ks value of 0 prevent the calculation of reliable Ka/Ks ratios. Results of this kind are uniformly labeled as “Na” and are not included in subsequent comparative analyses. The Ka/Ks values of the remaining collinear genes that could be calculated normally were all less than 0.6, indicating that they are all under the constraint of purifying selection.

### 3.7. Expression Analysis of Di19s in Sweet Potato and Its Two Diploid Relatives

#### 3.7.1. Expression Analysis in Different Tissues

**Figure 8:**
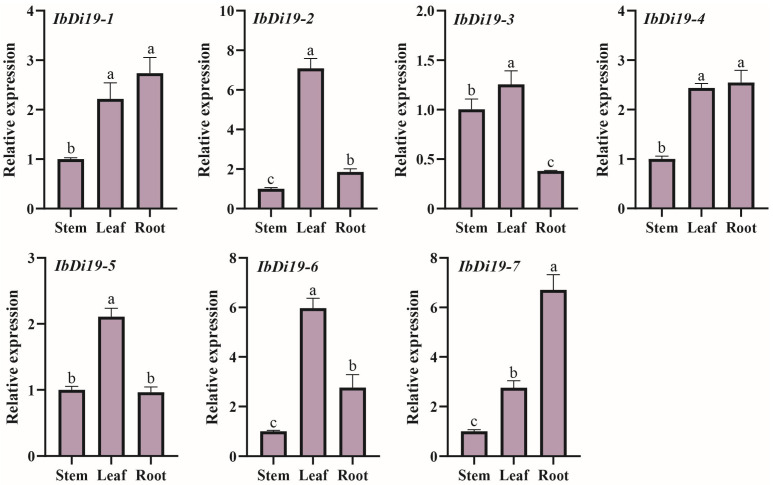
Expression analysis of *IbDi19s* in different tissues. Different lowercase letters denote statistically significant differences between groups.

The expression patterns of *IbDi19s* in the roots, stems, and leaves of in vitro plantlets were analyzed by RT-qPCR (Figure 8). The results showed that *IbDi19-1*, *IbDi19-4*, and *IbDi19-7* had the highest expression levels in the roots. *IbDi19-2*, *IbDi19-3*, *IbDi19-5*, and *IbDi19-6* had the highest expression levels in the leaves. Except for *IbDi19-3*, the expression levels of *IbDi19s* in the stem were relatively low.

**Figure 9:**
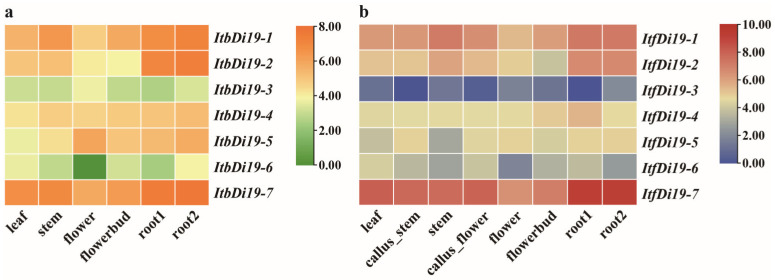
Expression analysis of *ItbDi19s* and *ItfDi19s* in different tissues. (**a**) Expression analysis of *ItbDi19s* in different tissues. (**b**) Expression analysis of *ItfDi19s* in different tissues.

An analysis was conducted on the expression patterns of *Di19s* in different tissues of *I. triloba* and *I. trifida* (Figure 9). The results showed that the expression patterns of *Di19s* in the two diploid relatives were similar. *Di19-7* had relatively high expression levels in various tissues, followed by *Di19-1*. The expression levels of *Di19-7*, *-1*, and *-2* in root1 and root2 were much higher than those of other *Di19* members in root1 and root2, and also much higher than their expression levels in most tissues. *ItbDi19-5* had relatively high expression levels in root2 and flower, and *ItbDi19-1* was highly expressed in flower bud and stem. *ItfDi19-2* was highly expressed in the stem.

#### 3.7.2. Expression Analysis in Response to Different Hormones

**Figure 10:**
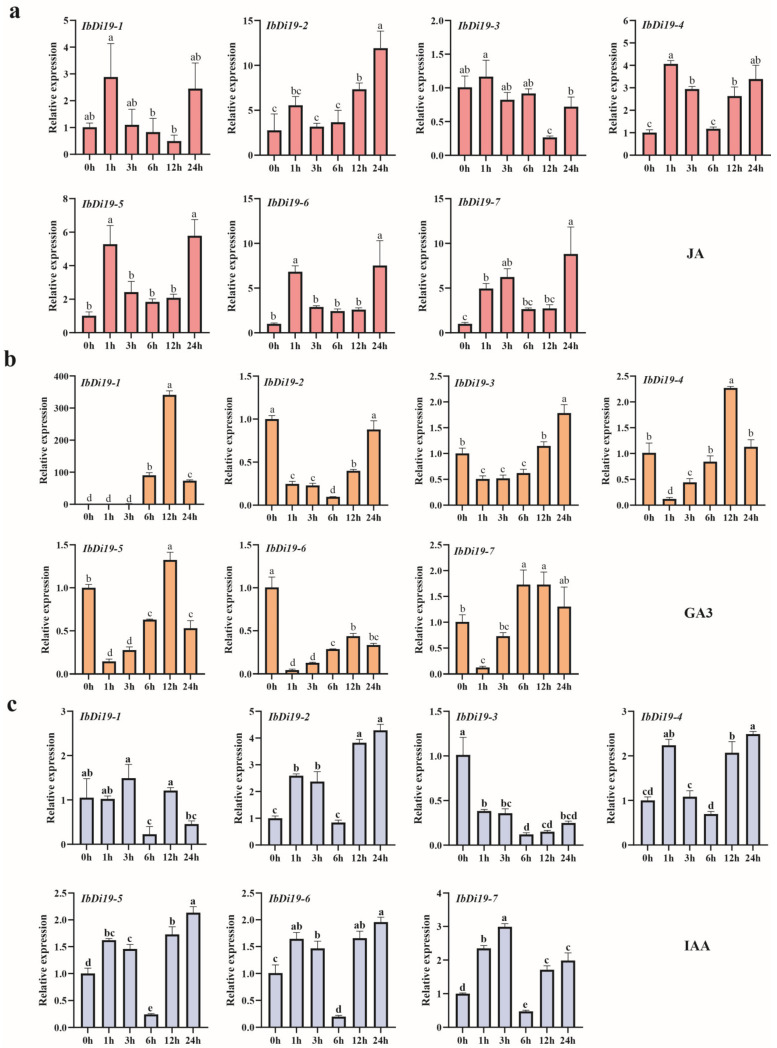
Expression analysis of *IbDi19s* under treatments of JA, GA3 and IAA. (**a**) Expression analysis of *IbDi19s* under treatments of JA. (**b**) Expression analysis of *IbDi19s* under treatments of GA3. (**c**) Expression analysis of *IbDi19s* under treatments of IAA. Different lowercase letters denote statistically significant differences between groups.

To explore the expression patterns of *IbDi19s* under different hormone responses, we performed RT-qPCR analysis of *IbDi19s* treated with JA, GA3, and IAA (Figure 10).

JA treatment induced distinct transcriptional responses of varying magnitudes among IbDi19 members. (Figure 10a). The expression patterns of *IbDi19-1*, *-4*, *-5*, *-6,* and *-7* were similar. Their expression levels increased to reach the first peak at 1 h or 3 h after JA treatment, then decreased, and finally increased again to reach the peak at 24 h after treatment. *IbDi19-2* reached the peak at 24 h after JA treatment, with the expression level approximately 4 folds that at 0 h. The expression level of *IbDi19-3* significantly decreased at 12 h and 24 h after JA treatment, but the overall change in expression level was very small.

There was no significant change in the expression level of *IbDi19-1* after 1 h and 3 h of GA3 treatment. (Figure 10b). It was significantly up-regulated at 6 h of GA3 treatment, with the expression level 89 folds that at 0 h. It showed the strongest response after 12 h of GA3 treatment, with the expression level 336 folds that at 0 h, reaching the peak. Finally, it was significantly down-regulated at 24 h of treatment, with the expression level 73 folds that at 0 h. *IbDi19-2*~*IbDi19-7* showed a trend of first significantly decreasing and then significantly increasing under GA3 treatment, but the overall fluctuation in expression level was very small compared with *IbDi19-1*.

The expression patterns of *IbDi19-2* and *IbDi19-7* were similar under IAA treatment (Figure 10c). Their expression levels first increased significantly, then decreased significantly, reaching the lowest level at 6 h of treatment, and finally increased significantly again. Although the remaining *IbDi19s* had significant expression in response to IAA treatment, the overall change in expression level was very small.

**Figure 11:**
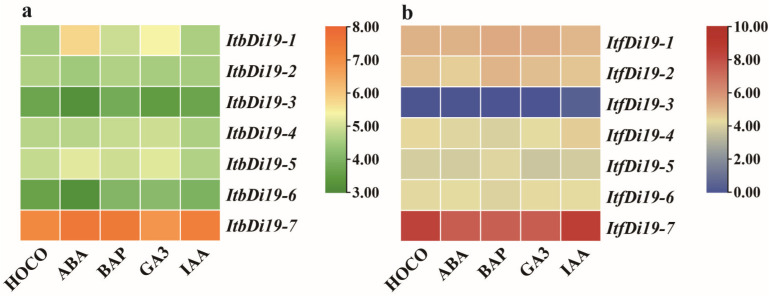
Expression analysis of *ItbDi19s* and *ItfDi19s* in response to different hormones. (**a**) Expression analysis of *ItbDi19s* in response to different hormones. (**b**) Expression analysis of *ItfDi19s* in response to different hormones. HOCO: hormone control experiment. The treatment concentrations of ABA, BAP, GA3, and IAA are 50 μM, 10 μM, 50 μM, and 10 μM, respectively.

Through the RNA-seq data of *I. triloba* and *I. trifida* under treatments of ABA, BAP, GA3 and IAA, the expression patterns of the two diploid relatives in response to different hormones were analyzed (Figure 11). In *I. triloba*, compared with the control group, *ItbDi19-7* was induced by ABA, BAP, and IAA, and inhibited by GA3 (Figure 11a). *ItbDi19-1* was induced by ABA and GA3, while other *ItbDi19s* were insensitive to hormone treatment. In *I. trifida*, compared with the control group, *ItfDi19-7* was induced by IAA, and inhibited by ABA, BAP, and GA3, while other *ItfDi19s* were insensitive to hormone treatment (Figure 11b).

#### 3.7.3. Expression Analysis Under Different Temperatures

**Figure 12:**
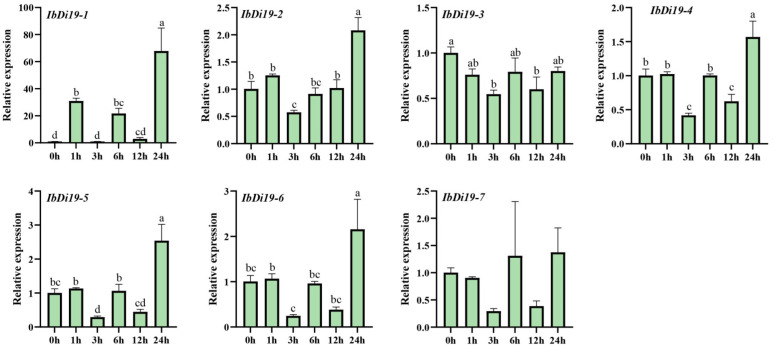
Expression analysis of *IbDi19s* under cold treatment. Different lowercase letters denote statistically significant differences between groups.

We analyzed the expression patterns of *IbDi19s* at 0 h, 1 h, 3 h, 6 h, 12 h, and 24 h under cold treatment at 4 °C (Figure 12). The expression of *IbDi19-1* was significantly up-regulated at 1 h and 6 h of treatment, with the expression levels being 31-fold and 22-fold that at 0 h, respectively. It was significantly down-regulated at 3 h and 12 h of treatment. At 24 h, it was strongly induced at 24 h under cold stress, reaching a 68-fold that of 0 h. The expression patterns of *IbDi19-2*, *-4*, *-5*, and *-6* were similar. Their expression levels significantly decreased at 3 h and significantly increased at 24 h. *IbDi19-7* did not show significant expression under cold treatment, and *IbDi19-3* was continuously down-regulated after cold treatment.

**Figure 13:**
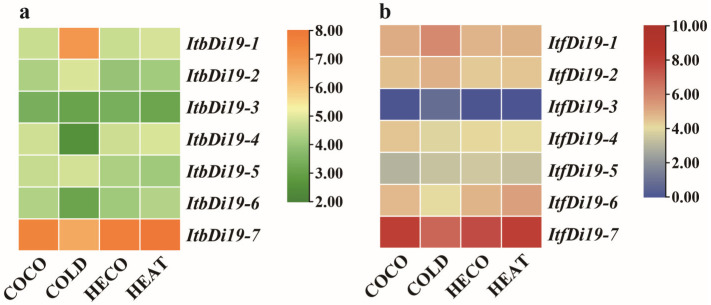
Expression analysis of *ItbDi19s* and *ItfDi19s* under low-temperature and high-temperature stresses. (**a**) Expression analysis of *ItbDi19s* under low-temperature and high-temperature stresses. (**b**) Expression analysis of *ItfDi19s* under low-temperature and high-temperature stresses. COLD: low-temperature stress experiment; HEAT: high-temperature stress experiment; COCO: cold control; HECO: heat control.

The RNA-seq data of the two diploid relatives under COLD and HEAT stresses were analyzed (Figure 13). Overall, the *Di19* gene family exhibited similar expression patterns in *I. triloba* and *I. trifida*. Compared with the COCO, *Di19-1* was induced under COLD, whereas *Di19-7* was severely inhibited under COLD. Notably, *ItbDi19-1* was strongly induced with a five-fold up-regulation (Figure 13a). Compared with the HECO, *Di19-7* was up-regulated under HEAT, while the expression levels of most *Di19s* changed little. In addition, *ItfDi19-6* displayed moderate up-regulation under HEAT (Figure 13b).

#### 3.7.4. Expression Analysis of Diploid Relatives Under Biotic and Abiotic Stresses

**Figure 14:**
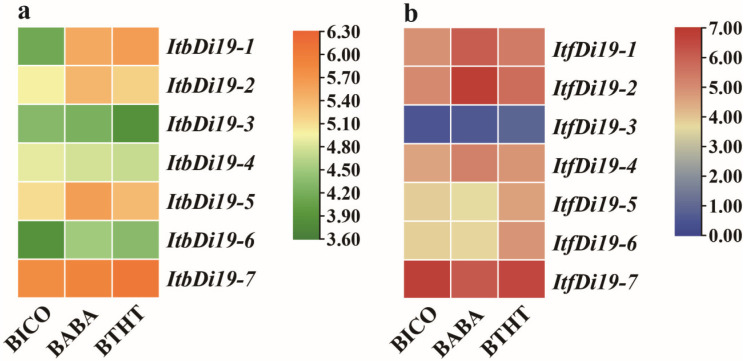
Expression analysis of *ItbDi19s* and *ItfDi19s* under biotic stress. (**a**) Expression analysis of *ItbDi19s* under biotic stress. (**b**) Expression analysis of *ItfDi19s* under biotic stress. BICO: biotic stress control; BABA: Beta-Aminobutyric Acid; BTHT: benzothiadiazole S-methylester.

To explore the responses of the two diploid relatives to biotic stress, we analyzed the RNA-seq data of *I*. *triloba* and *I*. *trifida* treated with two biotic stress elicitors: Beta-Aminobutyric Acid and benzothiadiazole S-methylester (Figure 14). In *I*. *triloba*, compared with the control group, *ItbDi19-1*, *-2*, *-5*, and *-6* were induced by BABA and BTHT, and *ItbDi19-7* was induced by BTHT (Figure 14a). Among them, *ItbDi19-1* showed the strongest response to the two plant disease-resistant inducers, with approximately three-fold up-regulation compared to the control. In *I*. *trifida*, compared with the control group, *ItfDi19-1*, *-2*, and *-4* were induced by BABA (Figure 14b). *ItfDi19-1* and *ItfDi19-2* were up-regulated approximately two-fold and three-fold, respectively, compared to the control. *ItfDi19-7* was inhibited by BABA and BTHT, and the remaining *ItfDi19s* were up-regulated to some extent under BTHT treatment.

**Figure 15:**
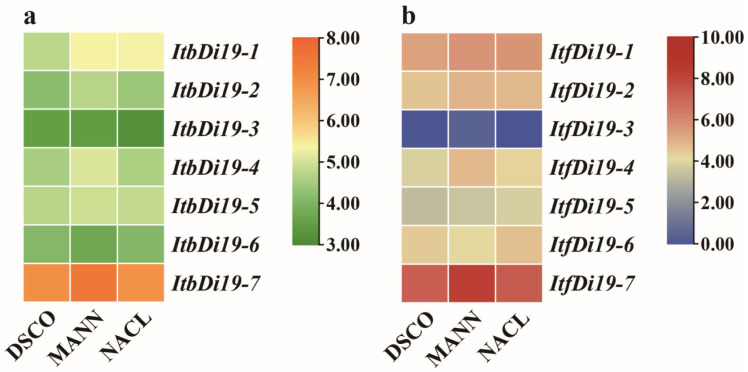
Expression analysis of *ItbDi19s* and *ItfDi19s* under drought and salt stresses. (**a**) Expression analysis of *ItbDi19s* under drought and salt stresses. (**b**) Expression analysis of *ItfDi19s* under drought and salt stresses. DSCO: drought and salt control experiment; MANN: mannitol drought stress experiment; NACL: NaCl salt stress experiment.

To explore the expression patterns of the two diploid relatives under drought and salt stresses, we analyzed their RNA-seq data from the MANN and the NACL experiment (Figure 15). *Di19* genes exhibited similar expression patterns in *I. triloba* and *I. trifida* under the mannitol drought stress experiment and the NaCl salt stress experiment. *Di19-7* showed the most intense response to the MANN experiment, while *Di19-6* was slightly inhibited. *Di19-1* was induced by the NACL experiment, while the expression levels of most *Di19s* changed only slightly under NACL treatment. *ItfDi19-3* was inhibited by the NaCl experiment.

## 4. Discussion

### 4.1. Evolution of Di19s in Sweet Potato and Its Two Diploid Relatives

In this study, seven members of the *Di19* gene family were identified in sweet potato and its two diploid relatives (Figure 1). This number is exactly the same as that of the *Di19* gene family members in several representative plants, such as *A. thaliana*, *O. sativa*, maize, barley, and poplar [14,16,18,22,25]. This indicates that the seven *Di19* members might be a highly conserved set of essential genes, representing the ancestral state of this family, and have been maintained stably by purifying selection during long-term evolution [55]. However, there are only six *Di19* members in grape, suggesting that a minor lineage-specific gene loss might have occurred [56,57]. In moso bamboo, there are ten *Di19* members, which may be related to its genome duplication and specific growth and development requirements [58,59]. In wheat, cotton, soybean, and peanut, the number has expanded to 15~18 [17,19,20,21]. This may be attributed to multiple whole-genome duplication or allopolyploidization events, and the duplicated genes were selectively retained or lost during domestication and adaptive evolution [60,61,62,63].

Di19 proteins exhibit numerous conserved features during the evolutionary process of sweet potato and its two diploid relatives. The *Di19* genes share identical distribution patterns in terms of chromosomal number and physical intervals in the two diploid relatives, indicating highly conserved chromosomal localization of *Di19s* between these two species (Figure 1). Comprehensive analysis of the intra-*Ipomoea* phylogenetic tree and the phylogenetic tree constructed with *A. thaliana* and *O. sativa* revealed that Di19 proteins from sweet potato and its two diploid relatives consistently clustered together, suggesting high sequence conservation of Di19 proteins among the three species (Figure 2 and Figure 3). All Di19s contain two characteristic domains. The number and type of predicted motifs in diploid-related species are consistent, indicating that the protein structure of Di19 is conserved (Figure 3b). In the collinearity and selection pressure analyses, multiple collinear gene pairs were identified both within and between the three species (Table 3 and Table 4, as well as Figure 6 and Figure 7). Moreover, the Ka/Ks ratios of these collinear gene pairs were all less than 0.6, suggesting that they were all under purifying selection constraints (Table 5).

Despite a high degree of conservation, significant lineage divergence has occurred in the *Di19* genes between sweet potato and its diploid relatives. In chromosomal localization, the *Di19-1*, *-2*, *-3*, and *-7* of the two diploids could all find corresponding copies on the related chromosomes of the sweet potato (Figure 1). However, *Di19-4*, *-5*, and *-6* located on adjacent chromosomes Chr10 and Chr11, respectively, in diploid, correspond to a single linkage group LG9 in sweet potato (Figure 1). This indicates that during the evolution of sweet potato from diploid ancestors to hexaploid, chromosomal rearrangement events might have occurred in the ancestral genome. As a result, the *Di19* gene clusters originally distributed on two chromosomes converged onto the same chromosome. Existing research has shown that hexaploid sweet potato is composed of two major subgenomes, B_1_ and B_2_ [64]. This species originated from triploids produced by crossing diploid B_1_ parents with tetraploid B_2_ parents, and then forming a hexaploid through whole genome duplication [64]. After the polyploidization event, extensive chromosomal rearrangements and fragment exchanges occurred between subgenomes, driving the reshaping of the sweet potato genome structure [64].

There is a significant divergence in the number of exons among the *Di19* family in sweet potato (Figure 3d). *IbDi19-2* and *IbDi19-7* maintain the conserved structure of five exons with their diploid relatives (Figure 3d). *IbDi19-4*, *-5*, and *-6* have been streamlined to four exons through exon loss, and the exons of *IbDi19-1* and *-3* have expanded to eight and seven, respectively (Figure 3d). In motif prediction, compared with diploid relatives, most Di19 proteins in sweet potato have experienced motif loss and substitution (Figure 3c). IbDi19-1 has lost motif 3, IbDi19-4, -5, and -6 have all lost motif 5, and motif 2 of IbDi19-2 has been replaced by motif 5 (Figure 3c). These differentiation characteristics might endow sweet potato with specific gene regulatory patterns and functions.

### 4.2. Role of IbDi19s in Abiotic Stress Responses in Sweet Potato

In RT-qPCR analysis, *IbDi19-1* showed a significant up-regulation in both cold treatment and GA3 treatment (Figure 10b and Figure 12). Its expression reached peak values at 24 h under cold treatment and 12 h under GA3 treatment (Figure 10b and Figure 12). Compared with the control, its expression was up-regulated by 68-fold and 336-fold, respectively, approximately 39-fold and 280-fold the expression levels of other *IbDi19s* during the same period (Figure 10b and Figure 12). *IbDi19-1* was particularly prominent among all the hormone treatments and members of the *Di19* family in this study. Since the maximum duration of cold treatment designed in this study was 24 h, and the expression level of *IbDi19-1* peaked at 24 h, it is possible that the expression of *IbDi19-1* would continue to increase with the extension of cold treatment time (Figure 12). *IbDi19-1* has the potential to continuously respond to cold treatment, which requires further experimental verification. In the COLD treatment, the expression of *ItbDi19-1* and *ItfDi19-1* was up-regulated approximately five-fold and two-fold, respectively, compared to the control (Figure 13). This was consistent with the expression trend of IbDi19-1 in the RT-qPCR analysis under cold treatment (Figure 12). The three genes are clustered in the same branch on the phylogenetic tree (Figure 2 and Figure 3).

In the analysis of promoter cis-acting elements, no classical gibberellin-responsive elements were predicted in *IbDi19-1* (Figure 4). Instead, multiple hormone-responsive elements for ABA, MeJA, SA, and IAA were present (Figure 4). It is speculated that its gibberellin response may not be achieved by directly binding to gibberellin pathway transcription factors through its own promoter. Instead, it might be regulated indirectly through cross-interactions of hormone pathways or upstream cascade indirect regulation. *IbDi19-1* was predicted to carry the low-temperature responsive element LTR (Figure 4). This element can mediate the transcriptional activation of genes by low-temperature signals, which explains the molecular reason for the strongly induced expression of this gene under cold stress at the cis-regulation level. The results of promoter prediction and RT-qPCR under low temperatures corroborated each other.

Both *ItbDi19s* and *ItfDi19s* have only five exons (Figure 3). However, the number of exons of *IbDi19-1* has reached eight during the evolutionary process, making it the member with the largest number of exons among all *IbDi19s* (Figure 3). In the Motif analysis, compared with the Di19 proteins of diploid relatives that clustered in the same branch, motif three of IbDi19-1 was lost (Figure 3). This increase in the number of exons and change in gene structure may enhance the gene’s stress response ability.

Research reports that gibberellin can promote processes such as seed germination, plant flowering, and release of bud dormancy in plants under low-temperature stress [65,66,67]. In common beans, RT-qPCR analysis revealed that different members of the *PvDi19* gene family exhibited varying degrees of induced expression under cold stress treatment [30]. The *TaDi19A* gene was identified from the transcriptome of wheat under low-temperature stress. Heterologous over-expression experiments confirmed that this gene can enhance the cold tolerance of *A. thaliana* [35]. The functional characteristics of *IbDi19-1* observed in this study are consistent with the trends of the above-mentioned previous research. In summary, *IbDi19-1* may play roles in the response to low-temperature stress and gibberellin signal transduction. However, this inference requires more experimental verification.

In the RT-qPCR analysis, most *IbDi19s* showed a significant up-regulation to a certain extent in response to JA treatment, while that of only *IbDi19-3* was significantly down-regulated. (Figure 10a). During the analysis of promoter cis-acting elements, 2~6 methyl jasmonate-responsive elements were predicted in the promoters of *IbDi19-1* to *IbDi19-6* (Figure 4). However, no such elements were detected in the promoter of *IbDi19-7* (Figure 4). *IbDi19-1* to *IbDi19-6*, which contain MeJA-responsive elements, may directly sense JA signals and activate transcription through promoter cis-elements. However, *IbDi19-7*, which lacks this element, can still respond to JA. This suggests that its response process may be independent of cis-elements and is achieved through indirect pathways.

### 4.3. Roles of Di19s in Growth, Development, Biotic and Abiotic Stresses in Two Diploid Relatives

In this study, the expression patterns of *ItbDi19s* and *ItfDi19s* in different tissues and under treatments of BABA, BTHT, ABA, BAP, GA3, IAA, COLD, HEAT, MANN, and NACL were analyzed (Figure 9 and Figure 11, as well as Figure 13, Figure 14 and Figure 15).

In the analysis of expression patterns in different tissues, it was found that the expression levels of *ItbDi19-7* and *ItfDi19-7* in various tissues were much higher than those of other *Di19* members (Figure 9). They might be housekeeping genes. The expression levels of *ItbDi19-7*, *-1*, *-2* and *ItfDi19-7*, *-1*, *-2* in root1 and root2 were much higher than those of other *Di19* members in root1 and root2, and also considerably higher than their own expression levels in other tissues (Figure 9). Additionally, *ItbDi19-5* had a relatively high expression level in root2 (Figure 9a). The swollen tuberous roots are the main products of sweet potato, which are rich in carbohydrates, vitamins, antioxidants, etc [4,5,6]. The homologous genes of these genes in sweet potato can serve as candidate genes for tuberous root development. *ItfDi19-1* is expressed at a consistently high level across multiple tissues, indicating that it plays a crucial role in the growth and development of *I. trifida* (Figure 9b). In addition, *ItfDi19-2* and *ItbDi19-1* are highly expressed in the stem, *ItbDi19-5* is highly expressed in the flower, and *ItbDi19-1* is highly expressed in the flower bud (Figure 9). This indicates that these genes may play important roles in the growth and development of the stem, as well as in the formation of floral organs and the process of reproductive development, respectively.

In response to biotic stress, compared with the control, the expression of *ItfDi19-1* and *ItfDi19-2* was up-regulated approximately two-fold and three-fold, respectively, under BABA treatment, and they were also induced under BTHT treatment (Figure 14). *ItbDi19-1* was induced under both BABA and BTHT treatments, with its expression up-regulated approximately three-fold compared to the control (Figure 14a). These three genes may be involved in the plant’s defense response to biotic stress mediated by BABA and BTHT.

In the response to abiotic stress, *ItbDi19-7* and *ItfDi19-7* may be house-keeping genes with high expression levels, and their basal expression levels are much higher than those of other family members (Figure 11, Figure 13 and Figure 15). This is consistent with the conclusion drawn from the previous study on the expression patterns in different tissues. Under hormone treatments, both *ItbDi19-7* and *ItfDi19-7* were inhibited by GA3 and induced by IAA (Figure 11). However, *ItbDi19-7* was induced by ABA and BAP, while *ItfDi19-7* was inhibited (Figure 11). This indicates that the two genes may be involved in different hormone-response pathways. The expression of both *ItbDi19-7* and *ItfDi19-7* was inhibited under COLD treatment, while it was induced under HEAT and MANN treatments (Figure 13 and Figure 15). The difference is that the magnitude of change in the expression level of *ItfDi19-7* was greater, suggesting that both genes are involved in the regulation of extreme high temperature and drought stress (Figure 13 and Figure 15). The expression level of *ItbDi19-1* was up-regulated to a certain extent when induced by ABA and GA3 (Figure 11b). In contrast, *ItfDi19-1* was insensitive to hormone treatments, with a relatively small change in expression level (Figure 11a). This indicates that the two genes participate in different hormone-response pathways, and *ItbDi19-1* may be associated with the responses to ABA and GA3.

## 5. Conclusions

In this study, seven members of the *Di19* gene family were identified in sweet potato and its two diploid relatives. The overall evolutionary pattern of this gene family is relatively conserved among the three species. Structural variations such as an increase in the number of exons, deletion, or substitution of conserved motifs occurred in IbDi19s in sweet potato. The expression patterns of *Di19s* in diploid relatives are highly conserved. *IbDi19s* are predominantly expressed in leaves and roots and respond significantly to exogenous jasmonic acid treatment. The expression level of the *IbDi19-1* was significantly up-regulated by 336-fold and 68-fold upon induction by gibberellin and low-temperature stress, respectively. It is speculated that *IbDi19-1* may be involved in the plant’s response to low-temperature stress and the regulation of the gibberellin signaling pathway. This study provides data support and candidate gene resources for deciphering the evolutionary patterns of the *Di19* gene family in sweet potato and investigating the molecular mechanisms of stress resistance of *Di19* genes.

## Figures and Tables

**Table 1:** Primer sequences for RT-qPCR analysis.

Primer	Primer Sequence
*IbActin_F*	AGCAGCATGAAGATTAAGGTTGTAGCACT
*IbActin_R*	GGAAAATTAGAAGCACTTCCTGTGAAC
*IbDi19-1_F*	CTTGCGACTGCAAACCTCTCAT
*IbDi19-1_R*	CCAACACTCCCAGCTTTCCTCT
*IbDi19-2_F*	CTGATCCATGGATTCGTTTCCCG
*IbDi19-2_R*	GGGCACATGAACTCTGGCCTAG
*IbDi19-3_F*	CTGGAACAATGCCACAGCAGC
*IbDi19-3_R*	TTCAGAATCCGAAGGGGGCAC
*IbDi19-4_F*	TTCTGGACCTCTCGTCTCGCC
*IbDi19-4_R*	CGGGCAAGGGAAATCAGGTCG
*IbDi19-5_F*	TGGATGCTGATCCTTGGTCGG
*IbDi19-5_R*	CGTCGACCATGTCAATTTCCTCG
*IbDi19-6_F*	TGGATGCTGATCCTTGGTCGG
*IbDi19-6_R*	CGTCGACCATGTCAATTTCCTCG
*IbDi19-7_F*	CTGATTTCTGGACCTCTCGCCT
*IbDi19-7_R*	GAAATCGGGTCGGGCCTCTT

Note: The CDS sequences of *IbDi19-5* and *IbDi19-6* are completely identical, and they share a pair of RT-qPCR primers. In the experiment, the amplification reaction systems of the two genes are independent of each other. Their expression levels are statistically analyzed separately, without any co-amplification interference.

**Table 2:** Sequence characteristics and physicochemical properties of IbDi19s.

Gene Name	Gene ID	Genomic Length (bp)	CDS * Length (bp)	Protein Size (aa)	MW * (kDa)	pI *	Instability Index	Gravy	Subcellular Locations
IbDi19-1	g5948	3558	777	258	29.03	4.81	79.97	−0.536	Nucleus
IbDi19-2	g6090	2801	507	168	19.06	4.45	75.62	−0.636	Nucleus
IbDi19-3	g33568	3292	975	324	36.06	6.53	56.66	−0.525	Nucleus
IbDi19-4	g34544	2922	645	214	23.97	5.79	61.29	−0.598	Nucleus
IbDi19-5	g35681	4243	612	203	22.63	5.31	54.61	−0.552	Nucleus
IbDi19-6	g35703	4241	612	203	22.63	5.31	54.61	−0.552	Nucleus
IbDi19-7	g59145	2425	720	239	26.99	5.53	62.12	−0.438	Nucleus

* CDS: coding sequence; MW: molecular weight; pI: isoelectric point.

**Table 3:** Intraspecific collinear genes of *Di19s* in sweet potato and its two diploid relatives.

Species	Segmental Duplication	
*I* *. batatas*	*IbDi19-2*	*g5507.t1*
	*IbDi19-3*	*g33527.t1*
	*IbDi19-5*	*IbDi19-6*
	*IbDi19-4*	*IbDi19-7*
*I* *. triloba*	*ItbDi19-3*	*ItbDi19-5*
	*ItbDi19-4*	*ItbDi19-7*
*I* *. trifida*	*ItfDi19-4*	*ItfDi19-7*
	*ItfDi19-3*	*itf10g19910.t1*

**Table 4:** Interspecific collinear genes of *Di19s* in sweet potato and its two diploid relatives.

*I* *. trifida-I* *. batatas*		*I* *. batatas-I* *. triloba*	
Gene1	Gene2	Gene1	Gene2
*ItfDi19-1*	*IbDi19-2*	*IbDi19-2*	*ItbDi19-1*
*ItfDi19-2*	*IbDi19-2*	*IbDi19-2*	*ItbDi19-2*
*ItfDi19-6*	*IbDi19-3*	*IbDi19-3*	*ItbDi19-6*
*ItfDi19-3*	*IbDi19-4*	*IbDi19-4*	*ItbDi19-3*
*itf10g19910.t1*	*IbDi19-4*	*IbDi19-4*	*ItbDi19-5*
*ItfDi19-4*	*IbDi19-6*	*IbDi19-6*	*ItbDi19-4*
*ItfDi19-3*	*IbDi19-7*	*IbDi19-7*	*ItbDi19-3*
*itf10g19910.t1*	*IbDi19-7*	*IbDi19-7*	*ItbDi19-5*

**Table 5:** Ka/Ks analysis of intraspecific and interspecific collinear gene pairs in sweet potato and its two diploid relatives.

Gene 1	Gene 2	Non-Synonymous Rate (Ka)	Synonymous Rate (Ks)	Substitution Rate Ratio (Ka/Ks)
*IbDi19-2*	*g5507.t1*	0.744	Na	Na
*IbDi19-3*	*g33527.t1*	0.016	0.044	0.370
*IbDi19-5*	*IbDi19-6*	0.000	0.000	NaN
*IbDi19-4*	*IbDi19-7*	0.108	0.696	0.155
*ItbDi19-3*	*ItbDi19-5*	0.096	0.679	0.141
*ItbDi19-4*	*ItbDi19-7*	0.104	0.592	0.175
*ItfDi19-4*	*ItfDi19-7*	0.104	0.548	0.190
*ItfDi19-3*	*itf10g19910.t1*	0.211	0.747	0.283
*ItfDi19-1*	*IbDi19-2*	0.005	0.009	0.559
*ItfDi19-2*	*IbDi19-2*	0.531	Na	Na
*ItfDi19-6*	*IbDi19-3*	0.018	0.044	0.414
*ItfDi19-3*	*IbDi19-4*	0.108	0.731	0.147
*itf10g19910.t1*	*IbDi19-4*	0.104	0.221	0.468
*ItfDi19-4*	*IbDi19-6*	0.000	0.007	0.000
*ItfDi19-3*	*IbDi19-7*	0.008	0.015	0.543
*itf10g19910.t1*	*IbDi19-7*	0.238	0.646	0.369
*IbDi19-2*	*ItbDi19-1*	0.005	0.000	Na
*IbDi19-2*	*ItbDi19-2*	0.582	Na	Na
*IbDi19-3*	*ItbDi19-6*	0.016	0.067	0.242
*IbDi19-4*	*ItbDi19-3*	0.108	0.698	0.154
*IbDi19-4*	*ItbDi19-5*	0.004	0.069	0.059
*IbDi19-6*	*ItbDi19-4*	0.004	0.022	0.190
*IbDi19-7*	*ItbDi19-3*	0.010	0.022	0.472
*IbDi19-7*	*ItbDi19-5*	0.103	0.685	0.151

## Data Availability

Data are contained within the article and Supplementary Materials.

## Supplementary Materials

The following supporting information can be downloaded at: https://www.mdpi.com/article/10.3390/genes17060712/s1. Table S1: Expression profiles of sweet potato and its two diploid relatives under different conditions.
